# Influence of KMnO_4_ Concentrationon Infrared Emissivity of Coatings Formed on TC4 Alloys by Micro-Arc Oxidation

**DOI:** 10.3390/ma10111301

**Published:** 2017-11-13

**Authors:** Guangrui Gao, Ying Li, Chaozhong Li, Dan Hu, Zhengxian Li, Zhengping Xi

**Affiliations:** 1 School of Metallurgy, Northeastern University, Shenyang 110819, China; 18792752738@163.com; 2 Research Institute of Corrosion and Protection, Northwest Institute for Nonferrous Metal Research, Xi’an 710016, China; lzxqy725@163.com; 3 Xi’an Surface Material Protection Co., Ltd., Xi’an 710016, China; pzhlchzh@163.com (C.L.); hudan139@sina.com (D.H.)

**Keywords:** micro-arc oxidation, TC4 alloys, infrared emissivity

## Abstract

Ceramic coatings with high emissivity were fabricated on TC4 alloys by micro-arc oxidation technique (MAO) in mixed silicate and phosphate electrolytes with varying KMnO_4_ addition. The microstructure, phase and chemical composition were characterized by scanning electron microscope (SEM), X-ray diffraction (XRD), and X-ray photoelectron spectroscopy (XPS), and the infrared emissivity of the MAO coatings was measured in a waveband of 5–20 μm. The results show that the thickness of the coatings increased with the addition of KMnO_4_, but the roughness of the coatings first decreased and then increased slightly due to the inhibitory effect of KMnO_4_ on Na_2_SiO_3_ deposition. The main phase composition of the coatings was anatase and rutile TiO_2_, amorphous form of SiO_2_ and MnO_2_. The infrared emissivity value of the coatings strongly depended on KMnO_4_ concentration, the coating formed at the concentration of 0.8 g/L KMnO_4_ reached the highest and an average of up to 0.87 was observed.

## 1. Introduction

High emissivity coatings have attracted intensive interest for use as energy-saving thermal protective coatings in high-temperature applications [1]. The high-emissivity coatings on titanium alloys have important application requirements in the field of electromagnetic propulsion, in particular. In general, the emissivity of metal oxides, especially for transition metal oxides, is higher than that of metals and their alloys [2]. MnO_2_, Fe_2_O_3_, CuO, Cr_2_O_3_, NiO, TiO_2_ and Ln_2_O_3_ [3,4] are usually used to prepare the related materials or coatings with high emissivity. Many surface treatment techniques have been used to deposit high-emissivity coatings, including brushing, plasma spraying [5], magnetron sputtering [6,7], electron beam physical vapor deposition etc. [8]. However, several widespread problems exist with these techniques, such as poor compactness and uniformity, low adhesion, and high cost.

Micro-arc oxidation (MAO), also called plasma electrolytic oxidation (PEO), is based on the conventional anodic oxidation of valve metals and their alloys in aqueous electrolyte solutions. However, it differs from the conventional anodic oxidation by its discharge feature, which is produced when the applied voltage exceeds the critical breakdown voltage of the insulated film [9]. It is well known that anodization is a process of forming an oxide film on the surface of valve metals under the action of the corresponding electrolyte and applied current. In the electrolysis process, the valve metals act as an anode, and oxygen is vigorously evolved on the valve metals to form an oxide film under the anodization conditions. The working area of the MAO process is transformed into the high-voltage discharge region compared with the common anodization. The discharge leads to localized high temperature and pressure in the corresponding discharge channel, which promotesthe thermoelectric reaction, thermochemical reactions, electrochemical reactions and simultaneous melting of the substrate and electrolyte, as well as the formation of the in situ oxide ceramic coating. MAO has been an effective surface treatment for greatly improving he hardness, wear resistance and the bonding strength of the ceramic coatings. Currently, the MAO technique is attracting huge attention because of its high productivity, economic efficiency and environmental friendliness. It is easy to design the composition of the coatings, and there are good prospects for the preparation of fine functional coatings [10,11]. In general, the spectral emissivity of MAO coatings is mainly determined by their chemical composition, surface condition and crystal structure. It should also be noted that MnO_2_ has high intrinsic infrared emissivity as a transition metal oxide. At present, little research has been done in this field, which has mainly been studied by the scholars at Harbin Institute of Technology. So it is of great importance to add KMnO_4_ in the electrolyte to form MnO_2_ in the coatings so as high spectral emissivity can be achieved for the coatings.

Commonly used electrolytes in MAO process include silicate, phosphate, and aluminate. The effects of silicate and phosphate electrolytes on the properties of micro-arc oxidation coatings have been studied by many researchers [10,11,12]. It is noteworthy that the application of aluminate electrolyte is limited, to a certain extent, due to its hydrolysis and poor stability. In this paper, the MAO coatings were prepared in mixed silicate, and phosphate electrolytes were added with varying KMnO_4_ concentrations, following which the effect factors on emissivity of the coatings were systematically studied. Additionally, the relationship between the properties of MAO coatings and the reaction mechanism are also discussed. Besides the experiment data, this work also provides a theoretical basis for the preparation of high-emissivity coatings by the MAO method.

## 2. Results and Discussion

### 2.1. Surface Morphology

Table 1 shows the thickness and surface roughness (Ra) of MAO coatings at different KMnO_4_ concentrations. Three samples were measured, and 10 points were selected on each sample for each measurement. It is obvious that the addition of KMnO_4_ can significantly reduce the roughness and increase the thickness of the coatings. The enhancement of the thickness is mainly due to the strong oxidability of KMnO_4_, which intensively promotes the oxidation reaction. However, the change of the coating thickness is not very obvious, and can be interpreted as the influence of the roughness on the thickness measurement.

**Figure 1:**
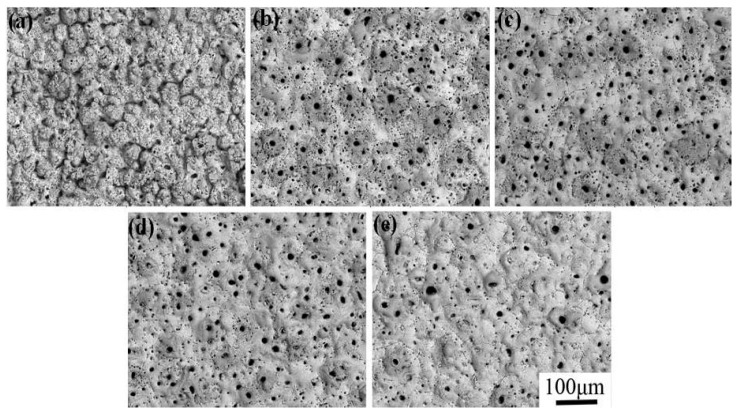
SEM images of MAO coatings at different KMnO_4_ concentration:(**a**) 0 g/L; (**b**) 0.8 g/L; (**c**) 1.6 g/L; (**d**) 2.4 g/L; (**e**) 3.2 g/L.

Figure 1 displays the surface morphology of MAO coatings at different KMnO_4_ concentrations. Obviously, all coatings exhibited typical porous structures with many micro pores on the surface, which are thought to be formed by the residues of the discharge in the MAO process [12,13]. However, there are great differences among their surface morphologies, as shown in Figure 1. It can be seen that there are many foam-like protrusions on the surface of the coating without the addition of KMnO_4_, while other surfaces are very smooth and compact [12]. The surface roughness of the MAO coatings was determined by their surface microstructures, especially the foam-like protrusions. The producing of foam-like protrusions caused by the electro deposition of silicate played the leading function in the MAO process [12]. The addition of KMnO_4_ inhibited the sintering reaction of silicate on the substrate surface, which resulted in the reduction of surface roughness by reducing the amount of silicate deposition. However, the surface roughness of the MAO coatings increased, subsequently, with the continued addition of KMnO_4_, which was due to the increase of film thickness. Some large discharge holes, and quite a lot of small discharge holes, were distributed on the surface of MAO coatings; their diameter exhibits huge differences, and the number of small discharge holes is much greater than that of the large ones for the same MAO coating. The diameter of the large holes showed no significant differences for the different coatings, which was ascribed to the two-step constant-pressure control method of oxidation. When the voltage was decreased, the current density and the oxidation also dropped, leading to the original discharge hole becoming smaller. The oxidation process was mainly caused by small discharge holes, which was beneficial to improving the density of the micro-arc oxidation film layer.

**Figure 2:**
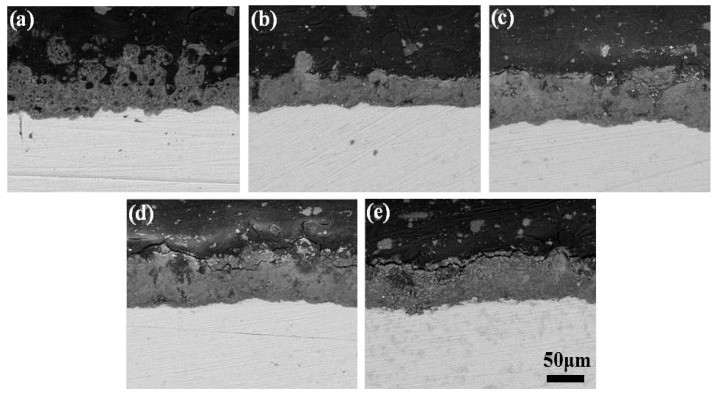
Cross-section morphology of MAO coatings at different KMnO_4_ concentration: (**a**) 0 g/L; (**b**) 0.8 g/L; (**c**) 1.6 g/L; (**d**) 2.4 g/L; (**e**) 3.2 g/L.

As can be seen from the cross-section morphology (Figure 2), the coatings and TC4 substrate could be clearly distinguished, and the discharge holes didn’t penetrate the whole film, but rather existed mainly in the surface layer, with the thickness of the coatings increasing steadily with the addition of KMnO_4_; the roughness of the MAO coatings without KMnO_4_ addition was the highest, which is consistent with the results shown in Table 1.

### 2.2. Phase Composition

**Figure 3:**
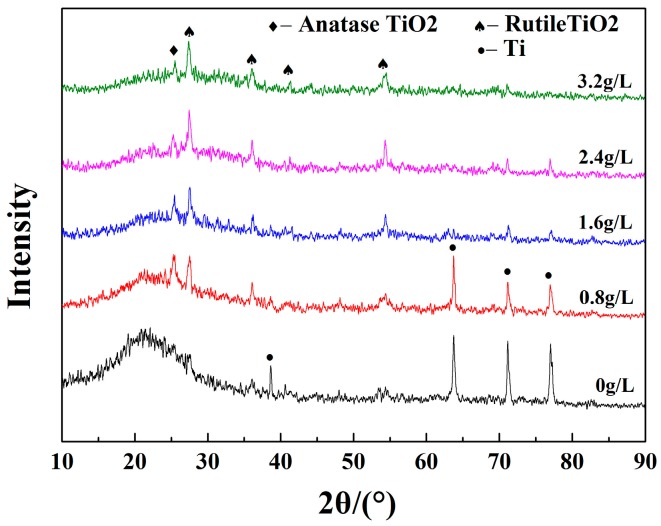
XRD patterns of MAO coatings at different KMnO_4_ concentrations.

The XRD diffraction patterns of the MAO coatings are shown in Figure 3; it can be seen from the figure that anatase-TiO_2_, rutile-TiO_2_ and Ti phases are present in the MAO coatings. The characteristic peak corresponding to Ti that appeared at high angles was due to the orientation growth of the crystal. The diffraction peaks of rutile TiO_2_ and anatase TiO_2_ became sharper with the increase in KMnO_4_ concentration, and therefore indicated that the crystallinity of the coatings changed to a better trend. In the course of solidification, TiO_2_ formed crystalline phase easily, and it was difficult for the amorphous phase to form by rapid cooling because of its high critical cooling rate. It can be inferred that Mn, P and Si elements existed in amorphous form, since the characteristic peaks of the compounds of Mn, P and Si elements were not detected. The amorphous phases were formed when the electrolyte was instantly cooled, and their existence was beneficial to improving the toughness and corrosion resistance of the MAO coatings.

The EDS results measured at the surface of the MAO coatings as shown in Figure 1 are summarized in Table 2. The results show that the coatings were composed of the elements O, Si, P, Ti and Mn. The chemical composition of the MAO coatings depended on substrate and electrolyte type. In the MAO process, the SiO_3_^2−^, PO_3_^−^ and MnO_4_^−^ that existed in the electrolyte were adsorbed onto the surface of the anode, and then entered into the discharge channel. The coatings were formed by these negative ions, and the melting and freezing of TiO_2_. The Mn content increased from 4.16 at.% to 9.32 at.% with the increase of KMnO_4_ concentration, while the silicon content obviously decreased to the contrary, implying that KMnO_4_ can significantly inhibit the deposition of silicon ions in the MAO process, which is consistent with the results of some relevant literature [14,15]. With the increase of KMnO_4_ addition, the content of P and Ti remained at a steady value, and there was no obvious change.

**Figure 4:**
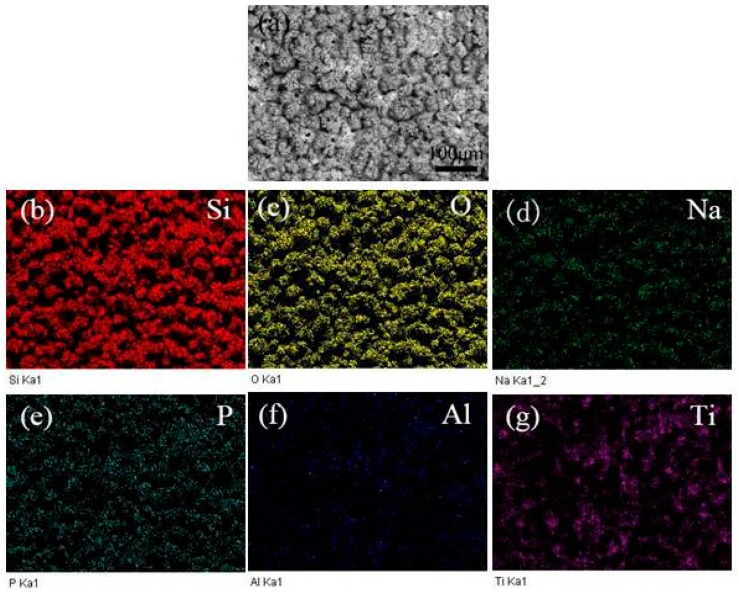
EDX mapping results of the sample prepared with 0 g/L concentration of KMnO_4_: (**a**) SEM morphology; (**b**) Si; (**c**) O; (**d**) Na; (**e**) P; (**f**) Al and (**g**) Ti.

**Figure 5:**
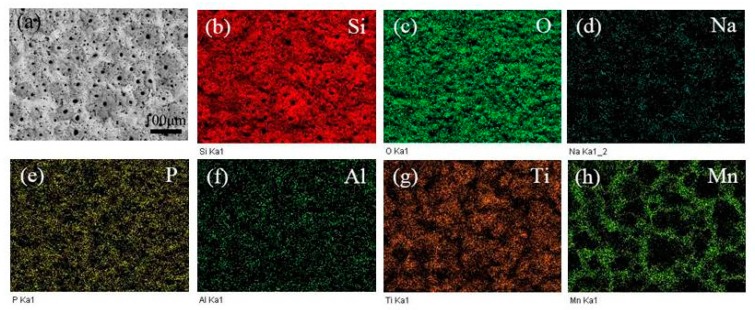
EDX mapping results of the sample prepared with 0.8 g/L concentration of KMnO_4_: (**a**) SEM morphology; (**b**) Si; (**c**) O; (**d**) Na; (**e**) P; (**f**) Al; (**g**) Ti and (**h**) Mn.

In order to study the distribution state of elements of the MAO coatings, the SEM/EDX images of the MAO coatings prepared at different KMnO_4_ concentration are presented in Figure 4 and Figure 5. According to the EDX mapping results in Figure 4, homogeneous distributions of the elements Si, O, Ti, P, Na, Al were observed on the surface of MAO coatings; they also reveal that the foam-like protrusions mainly consisted of silicate. Figure 5 shows that most of the Mn element was distributed around the discharge hole, and formed a network structure with the addition of KMnO_4_; the distribution state of the other elements exhibited no significant change.

**Figure 6:**
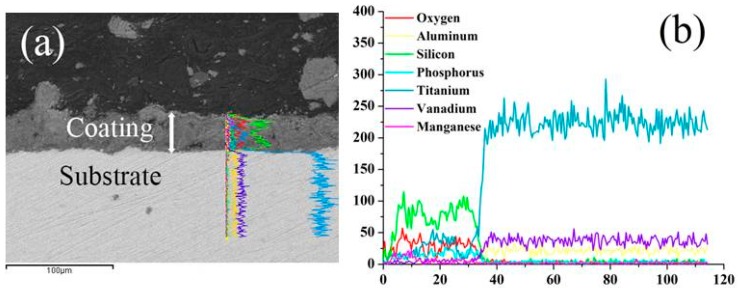
EDX analysis of the sample prepared with 0.8 g/L concentration of KMnO_4_: (**a**) Cross-sectional morphology of the MAO coating; (**b**) EDX elemental line scans.

The elemental distribution on the cross-section of the coating prepared at 0.8 g/L KMnO_4_ is shown in Figure 6. It clearly indicates that, with increasing coating thickness, the concentration of Ti, Al and V decreased gradually from the substrate, and existed stably in the coating, whereas the opposite trend was observed for Si, P and Mn elements, as these elements were in the electrolyte.

**Figure 7:**
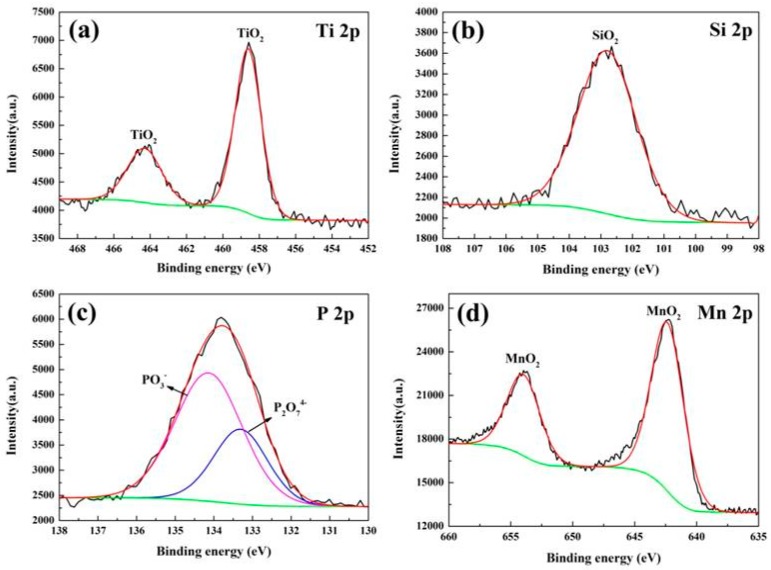
XPS spectra of MAO coatings prepared at KMnO_4_ concentration of 3.2 g/L: (**a**) Ti2p; (**b**) Si2p; (**c**) P2p; (**d**) Mn2p.

Since XRD is only able detect the existence of the crystals, we collected high-resolution XPS spectra of Ti, O, Si, P and Mn elements to confirm the chemical state of the elements in the coatings. The XPS spectra of the MAO coatings at KMnO_4_ concentration of 3.2 g/L are presented in Figure 7. As shown in Figure 7a, the peak of Ti2p at 458.7 eV was separately related with TiO_2_. In Figure 7b the spectrum of element Si2p exhibited one peak at 102.8 eV, suggesting that Si appeared in the tetravalent state Si^4+^, which was further explained by the fact that Si exists in the coating mainly in the form of SiO_2_ [16]. The P2p peak was well fitted at 134.1 eV and 133.3 eV in Figure 7c, which can be attributed to PO_3_^−^ and P_2_O_7_^4−^ [16,17,18,19]. The P_2_O_7_^4−^ could combine with Na^+^ to form Na_4_P_2_O_7_, and this conclusion is in good agreement with Hou’s report [19]. The spectrum of element Mn2p exhibited apeak at 642.4 eV (Figure 7d), implying that the Mn element existed in the form of MnO_2_ in the coatings [20].

### 2.3. Infrared Emissivity

According to the Kirchhoff laws, the emissivity of materials is equal to their absorption rate under the thermal equilibrium condition. Basic particles, such as electronics, atoms, and molecules, can release energy when vibration, rotation or transition occur. The emissivity of electromagnetic waves is the typical way of releasing the energy, and the majority of the electromagnetic radiation belongs to the infrared wavebands.

**Figure 8:**
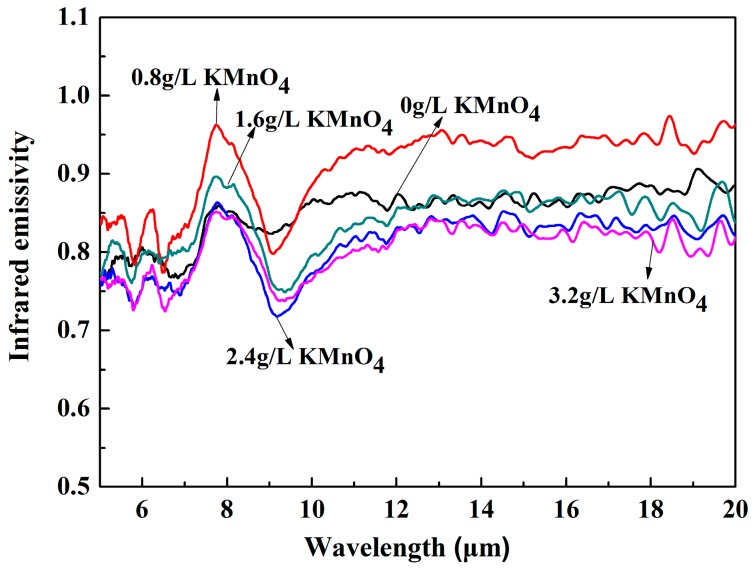
Infrared emissivity curves of the MAO ceramic coatings with different KMnO_4_ concentrations within a waveband of 5–20 μm.

The infrared emissivity curves for MAO ceramic coatings with a range of 5–20 μm relative to the blackbody are shown in Figure 8. It can be seen that the infrared emissivity of the MAO coatings first increased as the KMnO_4_ concentration increased, then the trend changed to a decrease. The maximum infrared emissivity was found for the coating with 0.8 g/L KMnO_4_, and this was significantly higher than the non-added coating, and the value was up to 0.92 greater than the 10 μm band. With greater addition of KMnO_4_, the emissivity of the coatings was significantly lower. Generally speaking, the emissivity was highly influenced by surface roughness and thickness, chemical composition and phase structure. In general, the emissivity increased with the increase of roughness and thickness [21,22]. However, these were not the leading factors in determining the change of emissivity, based on the test data of this research.

The highest infrared emissivity for the MAO coating with 0.8 g/L KMnO_4_ was primarily due to two mechanisms. First, the addition of KMnO_4_ promotes the formation of MnO_2_, which has high intrinsic emissivity, as a kind of transition metal oxide. Second, the doping of MnO_2_ causes crystal defects owing to the different ionic radius, which reduces the symmetry of lattice vibration and enhances the absorption bandwidth. In addition, the difference in electronic energy state leads to the formation of impurity energy levels at the defect, it should be noted that it would be beneficial to the transition of electrons and infrared absorption [23]. Meanwhile, its composition exhibited an amorphous form with structural properties of long-range disorder. Thus, local energy levels were formed between the lattice distortion region and the amorphous region, and it can also be deduced that the electrons were able to easily achieve transitions and promote infrared absorption in the short wavebands [24]. At the same time, factors including the high degree of confusion and large distortion coefficient of the amorphous structure, as well as the crystal defects caused by the doping effect, gave the coated atoms a strong polarity vibration, thus inducing photon radiation, leading to the increase of emissivity in the long wavebands [25].

Furthermore, it can be seen from the XRD results that the crystallinity of TiO_2_ was noticeably improved with the sustained addition of KMnO_4_; the crystal structure was more steady, leading to a subsequent reduction of infrared emissivity [26].

### 2.4. The Reaction Mechanism

The electrolyte contains the ions of H^+^, OH^−^, Na^+^, SiO_3_^2−^, PO_3_^−^ and MnO_4_^−^. Consequently, the following reactions in the MAO process occur in the electrolyte [12,17,18,19,20]:

a Cathodic reaction:
  2H^+^ + 2e^−^→H_2_↑ (1)
b Anodic reactions:
  4OH^−^ − 4e^−^→O_2_↑ + 2H_2_O (2)
  Ti + 4OH^−^ − 4e^−^→TiO_2_ + 2H_2_O (3)
  2MnO_4_^−^→MnO_4_^2−^ + MnO_2_ + O_2_↑ (4)
  2SiO_3_^2−^ − 4e^−^→2SiO_2_ + O_2_↑ (5)
  2PO_3_^−^ + 2OH^−^→P_2_O_7_^4−^ + H_2_O (6)

According to the literature research, Reaction (3) results in the oxidation of the TiC4 substrate, while the MnO_4_^−^ ions participated in a strong oxidation-reduction reaction in the MAO process due to its strong oxidability (Reaction (4)). The analysis considered that the (NaPO_3_)_6_ hydrolyzed into PO_3_^−^, and the PO_3_^−^ ions were able to be adsorbed onto the anode surface; at the same time, some PO_3_^−^ was able to be converted to pyrophosphate P_2_O_7_^4−^ under the high temperature and pressure of the MAO process (Reaction (6)). While Reactions (5) and (6) led to the deposition of electrolyte compounds in the MAO process.

## 3. Materials and Methods

TiC4 discs with 60 mm × 60 mm × 2 mm were used as the substrate. The surface of the discs was ground with SiC papers of up to 2000-grit, then rinsed in distilled water and ultrasonically cleaned in ethyl alcohol. The discs were used as anode, while a water-cooled electrolyser made of stainless steel served as the cathode. The MAO processes were carried out at 600 Hz for 40 min with a duty cycle of 30% using a 300 kW positive pulse power supply; the reaction temperature was maintained below 30 °C by adjusting stirring and the cooling-water systems. The electrolyte was prepared from the solution of Na_2_SiO_3_ (7 g/L); (NaPO_3_)_6_ (7 g/L), and the concentration of KMnO_4_ was 0 g/L, 0.8 g/L, 1.6 g/L, 2.4 g/L, 3.2 g/L, respectively. The micro arc oxidation treatment was controlled by the two-step constant pressure method. It was first oxidized at 500 V for 30 min, then the oxidation voltage was reduced to 480 V and oxidation was continued for 10 min.

Surface and cross-section morphologies of the MAO coatings, as well as their compositions, were studied by scanning electron microscopy (SEM, JSM6460, JEOL, Tokyo, Japan) with energy-dispersive X-ray spectrometry (EDX). Phase constituents of the coatings were examined with a D/max-rB automatic X-ray diffractometer (XRD, D/max-2200pc, RIGAKU, Tokyo, Japan) using a Cu Ka source. X-ray photoelectron spectroscopy (XPS, ESCALAB 250Xi, Thermo Fisher Scientific, Waltham, MA, USA) was utilized to analyze the compositions ofthe coating. Coating thickness was measured by eddy current thickness meter (TT 260, Time Company, Beijing, China). The roughness of the coatings was measured by current-based roughness gauge (TR-200, Time Company, Beijing, China). Infrared emissivity of the coatings was detected by a Fourier transform infrared spectrometer (FT/IR-6100, JASCO, Tokyo, Japan).

## 4. Conclusions

MAO processing was used to produce oxide coatings on TiC4 alloys in mixed silicate and phosphate electrolytes with different KMnO_4_ concentrations. The properties of the coatings were closely related to the contents of KMnO_4_. The MAO coatings presented a porous state, and the addition of KMnO_4_ was able to increase the thickness and reduce the surface roughness of the MAO coatings. Alternatively, the phase compositions of the MAO coatings exhibited little change. The TiO_2_ appeared as anatase and rutile TiO_2_ in the coatings, while the elements Si, P and Mn existed in amorphous form. The infrared emissivity exhibited the maximum value in the waveband of 5–20 μm when the concentration of KMnO_4_ was 0.8 g/L, mainly as a result of the doping effect and amorphous structure of MnO_2_. The emissivity didn’t increase or even drop when the KMnO_4_ content was further increased, because of the improvement of the crystallinity.

## Figures and Tables

**Table 1:** Thickness and surface roughness of MAO coatings at different KMnO_4_ contrations.

KMnO_4_ Concentration (g/L)	0	0.8	1.6	2.4	3.2
Thickness (μm)	35	33	40	41	46
standard deviation	0.415	0.621	0.543	0.572	0.608
Roughness (μm)	6.07	2.66	2.92	2.96	3.80
standard deviation	0.711	0.459	0.674	0.703	0.569

**Table 2:** Element content of MAO coatings at different KMnO_4_ concentrations.

KMnO_4_ Concetration (g/L)	Element Content of MAO Coatings (at.%)						
	O	Na	Al	Si	P	Ti	Mn
0	71.75	1.59	0.34	21.48	1.84	3.01	/
0.8	70.54	2.28	0.65	13.28	4.45	4.64	4.16
1.6	68.92	3.31	0.54	11.98	4.79	4.06	6.41
2.4	67.80	2.96	0.49	11.42	4.55	4.34	8.44
3.2	67.14	3.26	0.62	11.14	4.14	4.48	9.32
